# Supplementary material for: GREB1 induced by Wnt signaling promotes development of hepatoblastoma by suppressing TGFβ signaling
Source: Nat Commun. 2019 Aug 28;10:3882. doi: 10.1038/s41467-019-11533-x (PMC6713762; doi:10.1038/s41467-019-11533-x)
Supplement: Supplementary file 1 — Supplementary Information [file 41467_2019_11533_MOESM1_ESM.pdf]

Supplementary Information

Matsumoto et al.

GREB1 induced by Wnt signaling promotes development of hepatoblastoma by suppressing TGFβ signaling

Supplementary Figures 1-9 and associated legends.

Supplementary Tables 1-5.

Supplementary Fig. 1

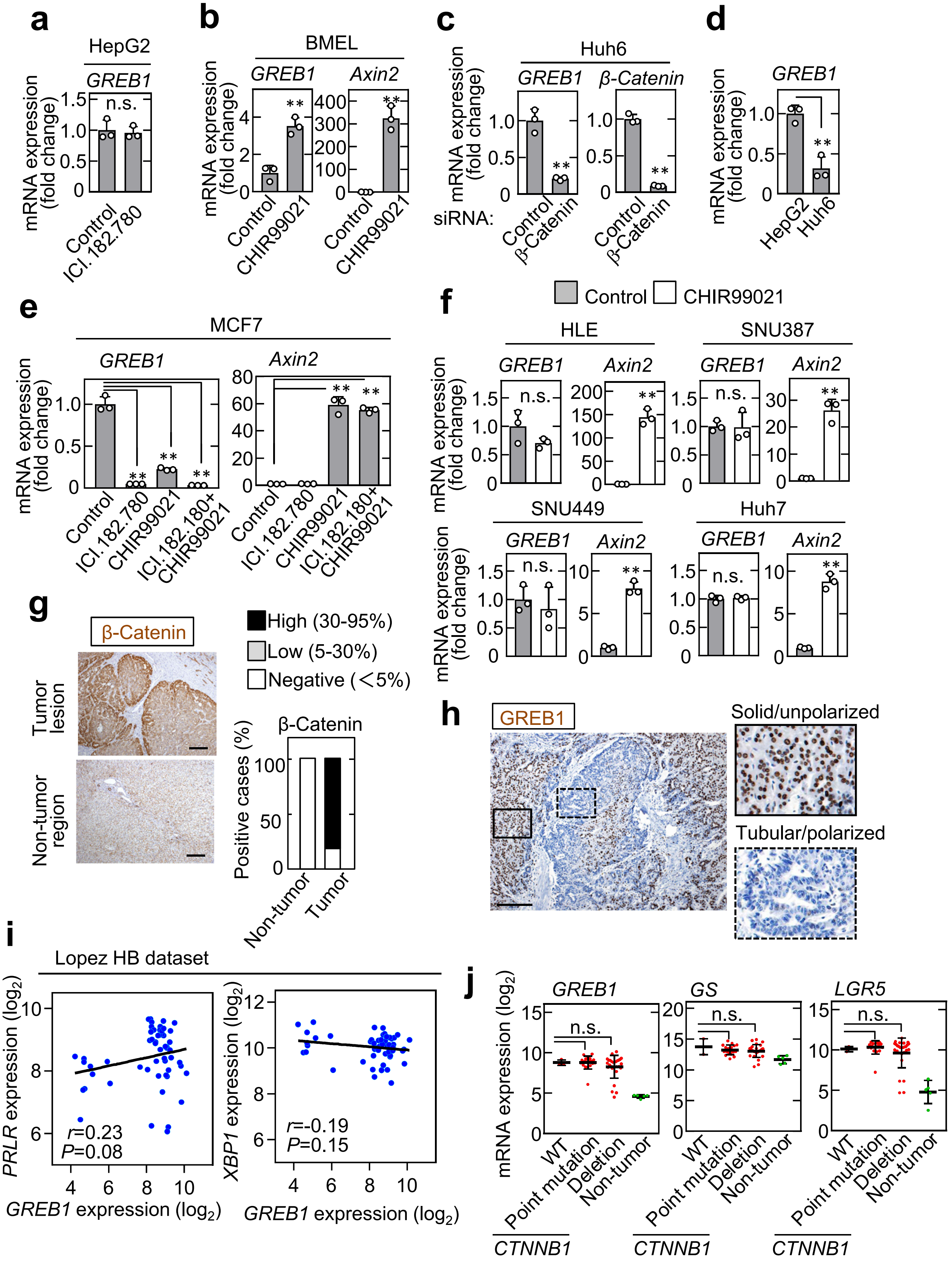

Supplementary Fig. 1| GREB1 is a downstream gene of Wnt/β-catenin signaling in HB cells.
a, HepG2 cells were treated with ICI.182.780 for 48 hours and real-time PCR experiments for *GREB1* mRNA expression were performed. Results are expressed as fold changes compared with control cells and are shown as means ± SD. **b.** Bipotential mouse embryonic liver (BMEL) cells were treated with 5 μM CHIR99021 for 24 hours and real-time PCR experiments for *GREB1* and *Axin2* mRNA expression were performed. **c**, Huh6 cells were transfected with control or *GREB1* #2 siRNA and real-time PCR experiments for the *GREB1* and *β-catenin* mRNA expression were performed. **d,** Real-time PCR experiments of HepG2 and Huh6 cells for GREB1 mRNA expression were performed. **e,** MCF7 cells were untreated or treated with 10 μM ICI.182.780, 5 μM CHIR99021, or their combination for 24 hours. Real-time PCR experiments for *GREB1* and *Axin2* mRNA expression were performed. **f,** HLE, SNU387, SNU449, or Huh7 cells were treated with 5 μM CHIR99021 for 24 hours and real-time PCR experiments for *GREB1* and *Axin2* mRNA expression were performed. **g,** HB tissues (n=11) were stained with anti-β-catenin antibody and hematoxylin. Percentages of β-catenin-positive cases in the tumor lesions and non-tumor regions are shown. Areas that stained positive for β-catenin were classified as indicated. **h,** HB tissues (n=11) were stained with anti-GREB1 antibody and hematoxylin. Solid and dashed squares show enlarged images. **i,** Scatter plots showing correlation between target genes of estrogen receptor (Y-axis) and *GREB1* gene expression (X-axis) were obtained from 55 patient samples in the public mRNA profile dataset of HB. The solid line indicates linear fit. *r* indicates Pearson's correlation coefficient. *r* value and *P* value were calculated with GraphPad Prism 7. **j**, *GREB1,GS,* and *LGR5* gene expression were analyzed in 5 non-tumor and 50 HB cases with WT (n=3), point mutation (n=19), or deletion (n=28) of exon3 and 4 of the *CTNNB1* gene, which were obtained from public mRNA profile dataset of HB. Results shown are scatter plots with means ± SD. n.s., not significant. ** $P<0.01$, *t* test in **a**-**d**, and **f**; ANOVA and post hoc test in **e** and **j**. Scale bars in **g** and **h,** 200 μm.

Supplementary Fig. 2

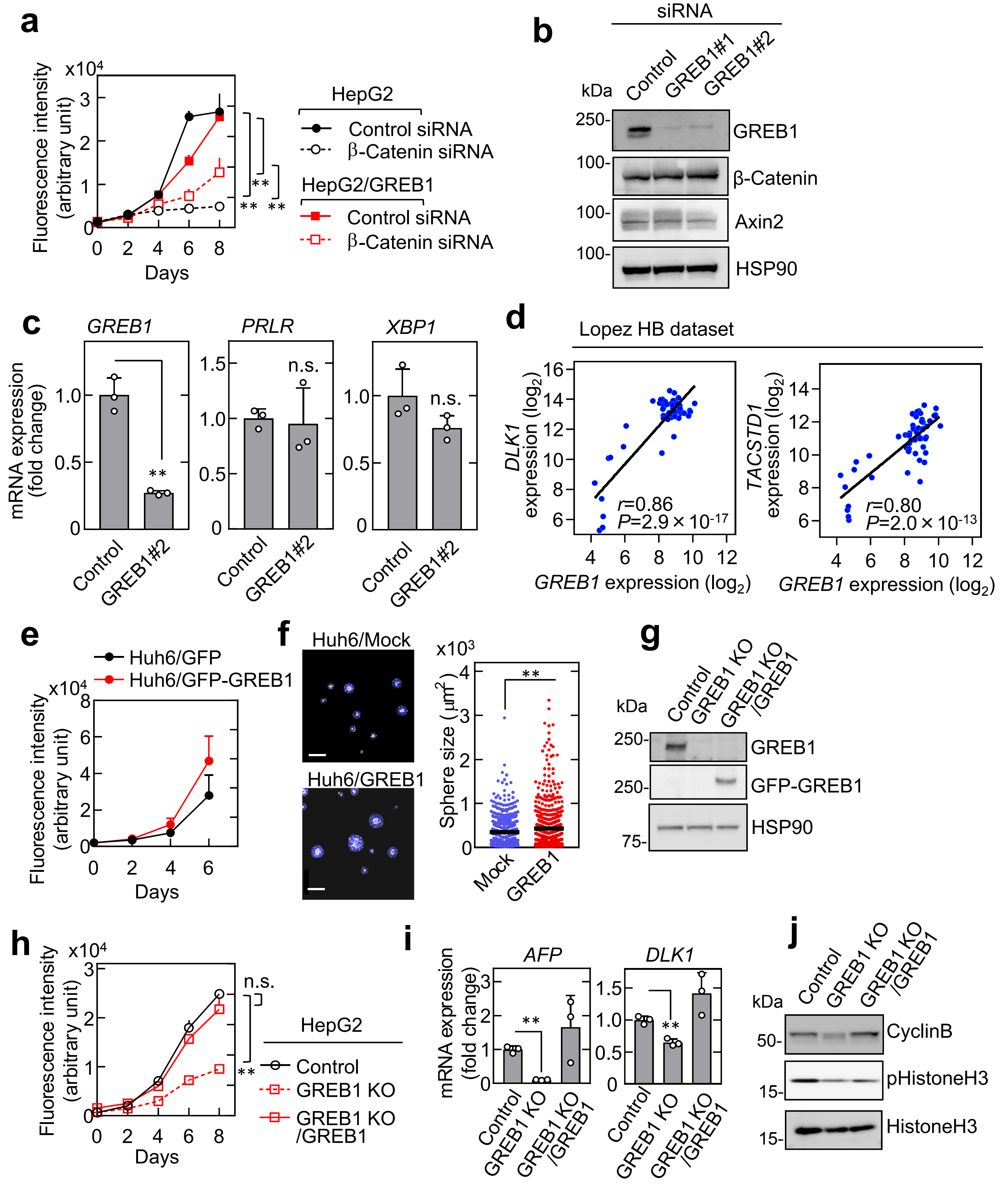

Supplementary Fig. 2| GREB1 promotes proliferation of HB cells *in vitro.*

a, Control or GFP-GREB1 expressing HepG2 cells were transfected with control or β-catenin siRNAs and cultured on a two-dimensional (2D) plastic dish for the indicated numbers of days. Relative cell numbers were quantified using the Cyquant assay. Results are shown as means ± SD. **b,** HepG2 cells were transfected with control or two independent *GREB1* siRNAs and the lysates were probed with anti-GREB1, anti-β-catenin, anti-Axin2, and anti-HSP90 antibodies. **c**, HepG2 cells were transfected with control or *GREB1*#2 siRNA and real-time PCR experiments for indicated mRNA expression were performed. Results are expressed as fold changes compared with control cells and are shown as means ± SD. **d**, Scatter plots showing a correlation between expression of marker genes of HB (*DLK1* and *TACSTD1*) (Y-axis) and *GREB1* gene (X-axis) were obtained from the public mRNA profile dataset of HB. The solid line indicates a linear fit; r indicates Pearson's correlation coefficient. *r* value and *P* value were calculated with GraphPad Prism 7. **e**, Huh6 cells expressing GFP or GFP-GREB1 were cultured on a 2D plastic dish for the indicated numbers of days, and relative cell numbers were quantified using the Cyquant assay. **f**, Huh6 cells expressing mock or GREB1 were cultured for 6 days in 3D Matrigel. Cells were then stained with phalloidin and Hoechst33342, and areas of spheres were calculated. Results are shown as scatter plots with means (n > 500). **g**, Cell lysates from control, GREB1 knockout (KO), or GREB1 KO expressing GFP-GREB1 HepG2 cells were probed with indicated antibodies. **h**, Control, GREB1 KO, or GREB1 KO expressing GFP-GREB1 HepG2 cells were cultured on a 2D plastic dish for the indicated numbers of days. Relative number of cells was quantified using the Cyquant assay. **i**, Real-time PCR analyses for expression of the indicated mRNAs of control, GREB1 KO, or GREB1 KO expressing GFP-GREB1 HepG2 cells were performed. **j**, Cell lysates from control, GREB1 KO, or GREB1 KO expressing GFP-GREB1 HepG2 cells were probed with the indicated antibodies. n.s., not significant. $P<0.01$, *t* test in **c** and **f**; ANOVA and post hoc test in **a**, **h**, and **i**. Scale bar in **f**, 100 μm.

Supplementary Fig. 3

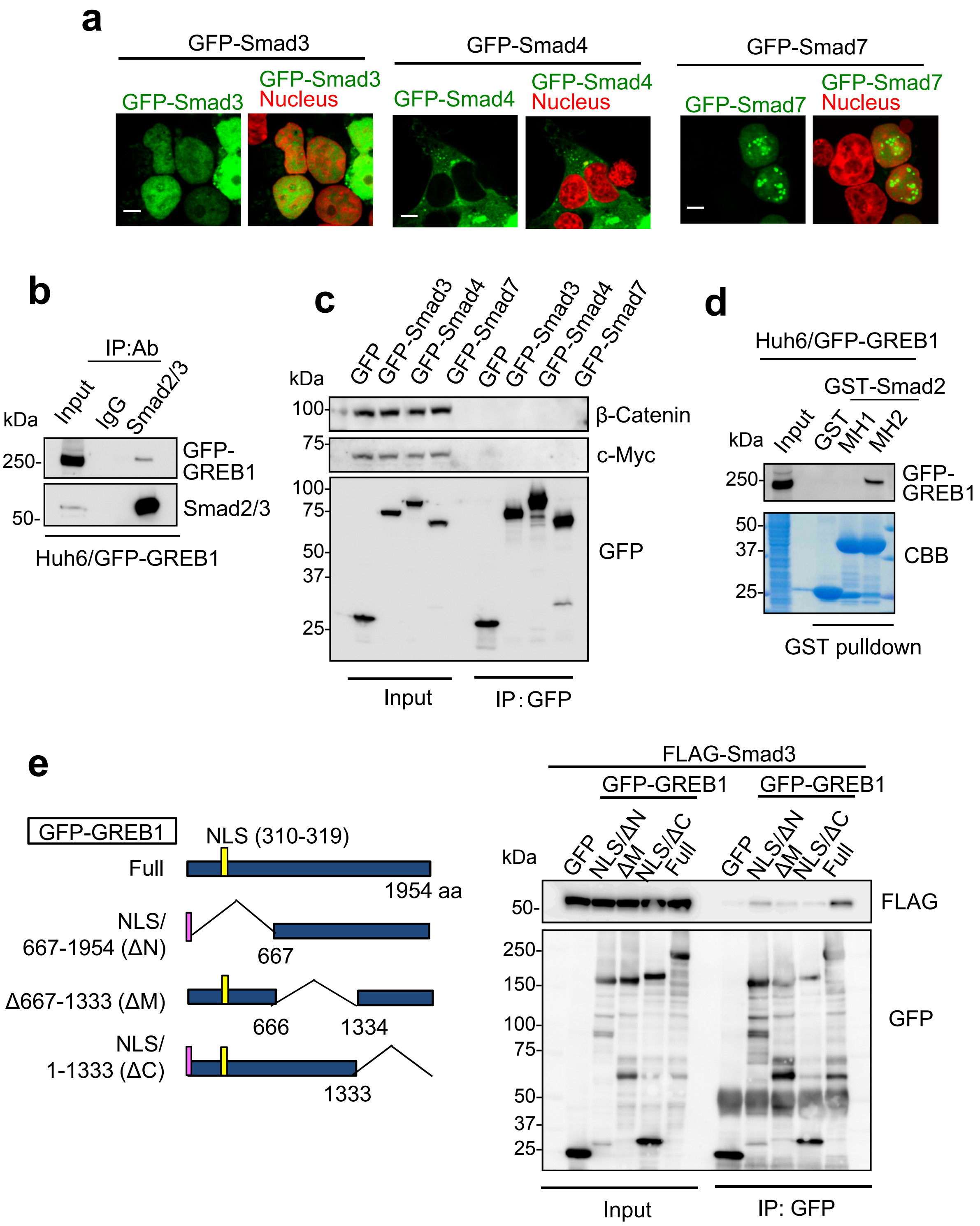

Supplementary Fig. 3| GREB1 interacts with Smad2/3.

a, X293T cells expressing the indicated proteins were stained with anti-GFP antibody and Hoechst33342. **b,** Lysates of Huh6 cells expressing GFP-GREB1 were immunoprecipitated with anti-Smad2/3 antibody and the immunoprecipitates were probed with the indicated antibodies. **c,** Lysates of X293T cells expressing the indicated proteins were immunoprecipitated with anti-GFP antibody, and the immunoprecipitates were probed with the indicated antibodies. **d**, Lysates of Huh6 cells expressing GFP-GREB1 were precipitated with recombinant GST, GST-Smad2/MH1, or GST-Smad2/MH2 and the precipitates were probed with anti-GFP antibody and stained with Coomassie Brilliant Blue. **e**, Schematic representation of the GFP-GREB1 (667-1954/ΔN), GFP-GREB1(Δ667-1333/ΔM), and GFP-GREB1(1-1333/ΔC) are shown. Lysates of X293T cells expressing the indicated proteins were immunoprecipitated with anti-GFP antibody and the immunoprecipitates were probed with the indicated antibodies. Scale bars in **a**, 5 µm.

Supplementary Fig. 4

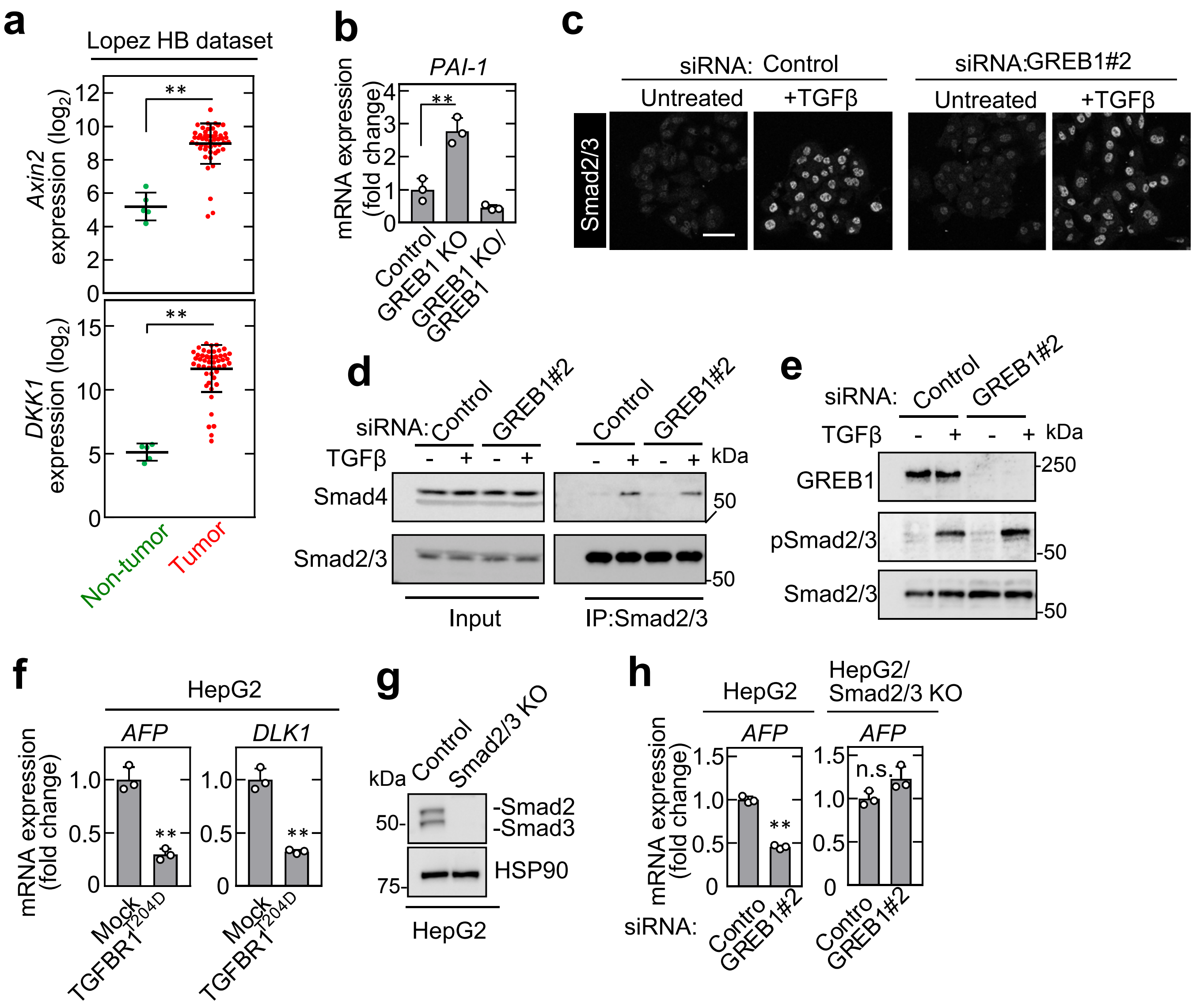

Supplementary Fig. 4| GREB1 regulates TGFβ signaling independently of TGFβ-induced phosphorylation, binding to Smad4, and nuclear localization of Smad2/3.

a, *Axin2* and *DKK1* gene expression, which were obtained from public mRNA profile dataset of HB, was analyzed. Results are shown as scatter plots with means ± SD. **b**, real-time PCR analyses for *PAI-1* mRNA expression of control, GREB1 knockout (KO), or GREB1 KO expressing GFP-GREB1 HepG2 cells were performed. Results are expressed as fold-changes compared with control cells and are shown as means ± SD. **c**, HepG2 cells transfected with control or *GREB1* #2 siRNA were untreated or treated with 10 ng/ml TGFβ1 for 30 min. Cells were stained with anti-Smad2/3 antibody. **d**, Lysates of HepG2 cells transfected with control or *GREB1* #2 siRNA and untreated or treated with 10 ng/ml TGFβ1 for 30 min were immunoprecipitated with anti-Smad2/3 antibody. The immunoprecipitates were probed with anti-Smad4 and anti-Smad2/3 antibodies. **e**, HepG2 cells transfected with control or *GREB1* #2 siRNA were untreated or treated with TGFβ. The lysates were probed with the indicated antibodies. **f**, Real-time PCR analyses for indicated mRNA expression of HepG2 cells expressing mock or $TGFBR^{T204D}$ were performed. **g**, Lysates from control or Smad2/3 knockout (KO) HepG2 cells were probed with anti-Smad2/3 and anti-HSP90 antibodies. **h**, Control or Smad2/3 KO HepG2 cells were transfected with control or *GREB1* #2 siRNA, and real-time PCR analyses for *AFP* mRNA expression of these cells were performed. n.s., not significant. $P<0.01$, *t* test in **a**, **f**, and **h**; ANOVA and post hoc test in **b**. Scale bar in **c**, 50 μm.

Supplementary Fig. 5

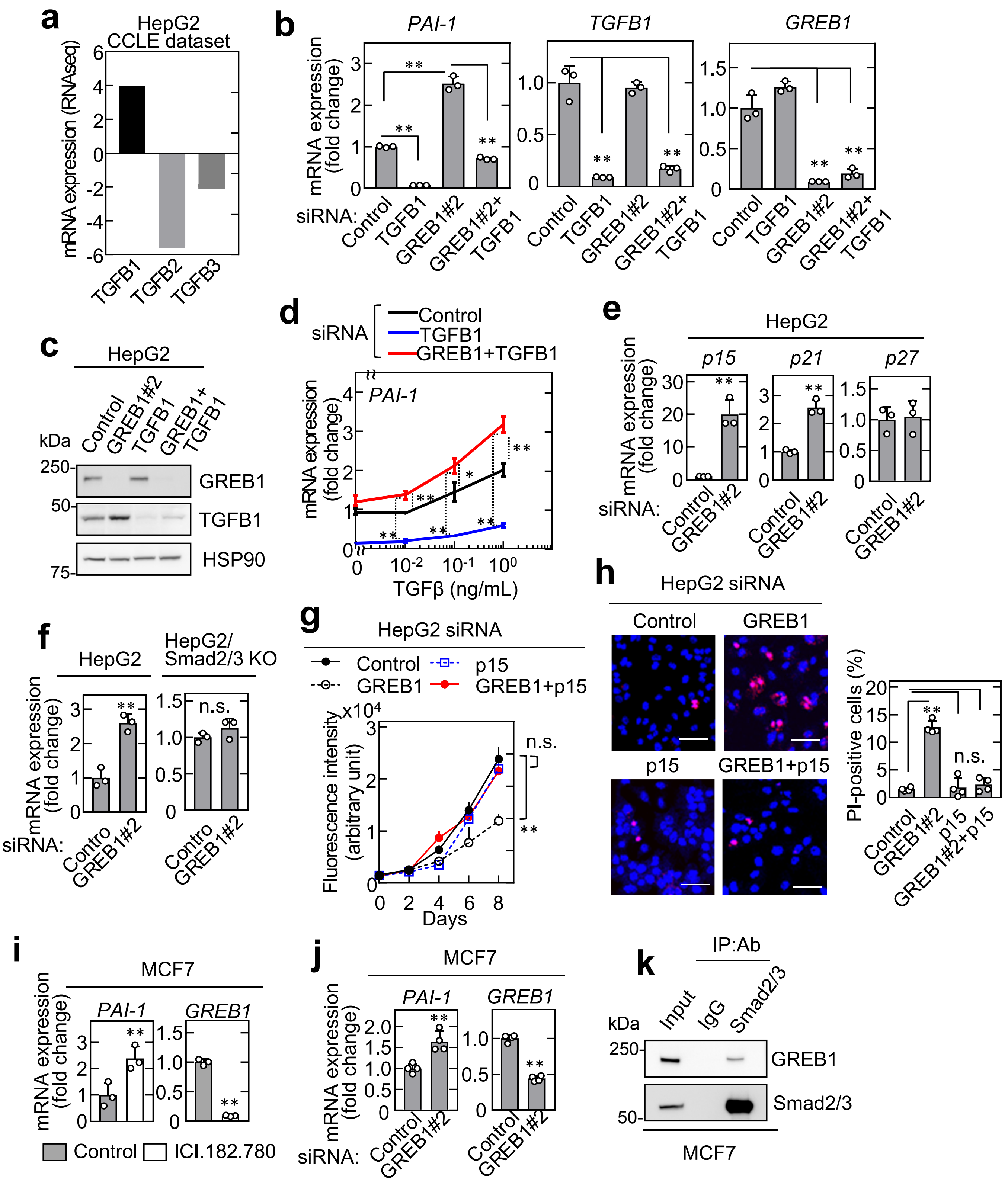

Supplementary Fig. 5| GREB1 regulates cell proliferation and survival through the inhibition of TGFβ-Smads-p15 pathway.

a, Expression levels of *TGFB1*, *TGFB2*, and *TGFB3* mRNA in HepG2 cells were obtained from Cancer Cell Line Encyclopedia (CCLE) RNA-seq dataset. **b**, HepG2 cells were transfected with siRNAs for control, *TGFB1, GREB1*, or *GREB1* and *TGFB1* for 72 hours and real-time PCR experiments for expression of the indicated mRNAs were performed. Results are shown as means ± SD. **c**, HepG2 cells were transfected with siRNAs for control, *GREB1*, *TGFB1,* or *GREB1* and *TGFB1* for 72 hours and the lysates were probed with anti-GREB1, anti-TGFB1, and anti-HSP90 antibodies. **d**, HepG2 cells were transfected with the indicated siRNAs, and untreated or treated without TGFβ1 at 0.01, 0.1, or 1 ng/ml for 4 hours. Real-time PCR experiments for *PAI-1* mRNA expression of these cells were performed. **e,** HepG2 cells were transfected with control or *GREB1*#2 siRNA and real-time PCR experiments for indicated mRNA expression were performed. **f,** Control or Smad2/3 KO HepG2 cells were transfected with control or *GREB1* #2 siRNA, and real-time PCR experiments for expression of *p15* and *PAI-1* mRNAs were performed. **g,** HepG2 cells were transfected with control, *GREB1*#2, *p15*, or *GREB1#2* and *p15* siRNAs were cultured on a 2D plastic dish for the indicated numbers of days. Relative number of cells was quantified using the Cyquant assay. **h**, HepG2 cells were transfected with control, *GREB1*#2, *p15*, or *GREB1#2* and *p15* siRNAs were cultured in medium with 0.1% FBS for 48 hours, and the lysates were probed with the indicated antibodies. **i**, MCF7 cells were treated with ICI.182.780 for 48 hours and real-time PCR analyses for expression of *PAI-1* and *GREB1* mRNAs were performed. **j**, MCF7 cells were transfected with control or *GREB1 #2* siRNA, and real-time PCR analyses for expression of *PAI-1* and *GREB1* mRNAs were performed. **k,** Lysates of MCF7 cells were immunoprecipitated with anti-Smad2/3 antibody and the immunoprecipitates were probed with anti-GREB1 and anti-Smad2/3 antibodies. n.s., not significant. ** $P<0.01$; * $P<0.05$, *t* test in **e**, **f**, **i**, and **j**; ANOVA and post hoc test in **b**, **d**, **g**, and **h**. Scale bar in **h**, 50 μm.

Supplementary Fig. 6

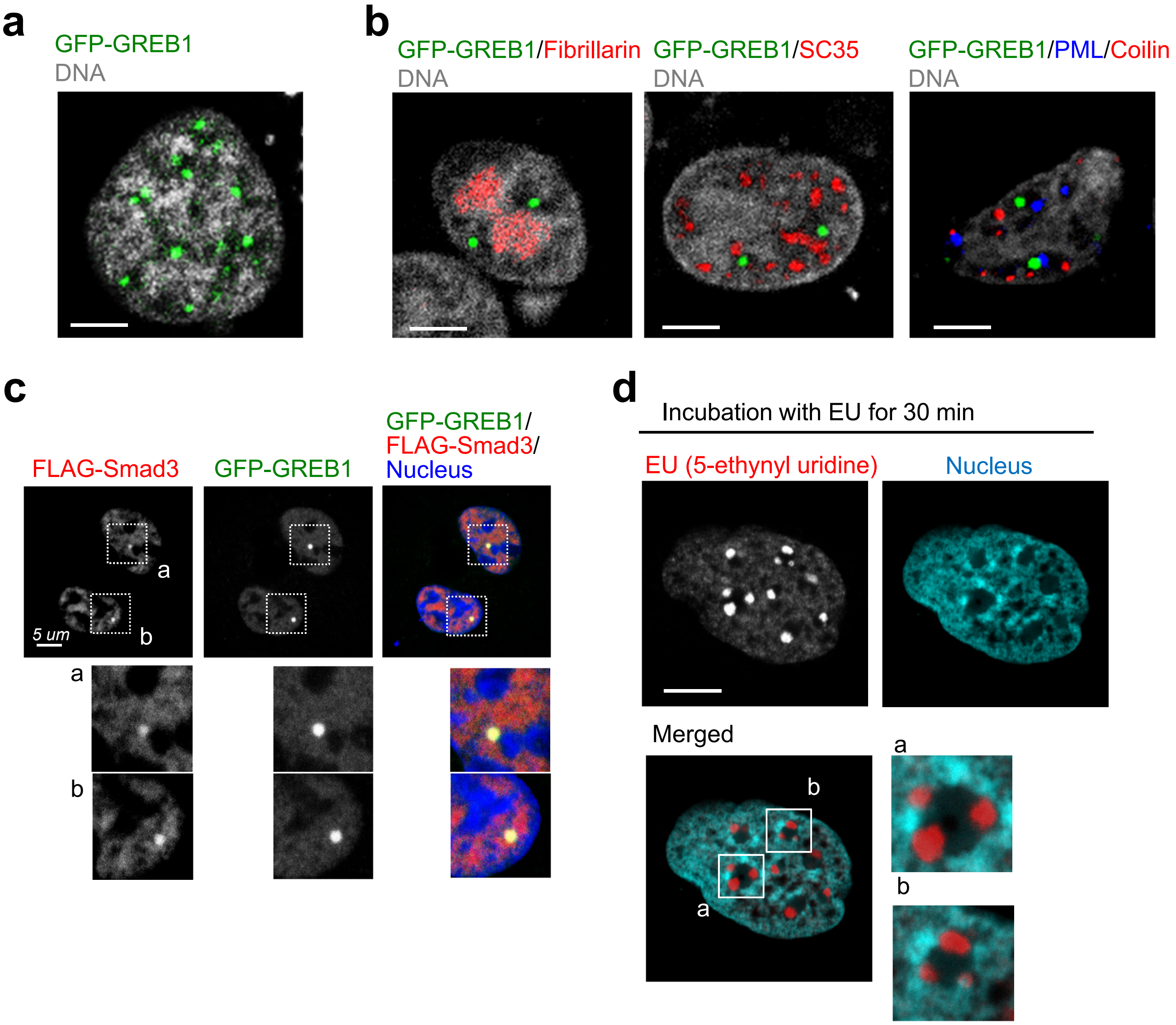

Supplementary Fig. 6| GREB1 co-localizes with Smad2/3 in the interchromatin space.
a, HepG2 cells expressing GFP-GREB1 were stained with anti-GFP antibody and Hoechst33342. **b**, HepG2 cells expressing GFP-GREB1 were stained with the indicated antibodies and Hoechst33342. **c**, HepG2 cells expressing GFP-GREB1 and FLAG-SMAD3 were stained with anti-GFP or anti-FLAG antibody and Hoechst33342. The regions in the Dashed squares are shown enlarged. **d**, HepG2 cells were incubated with EU for 30 min before fixation. The cells were stained with Hoechst33342. The regions in the Solid squares are shown enlarged. Scale bars, 5 μm.

Supplementary Fig. 7

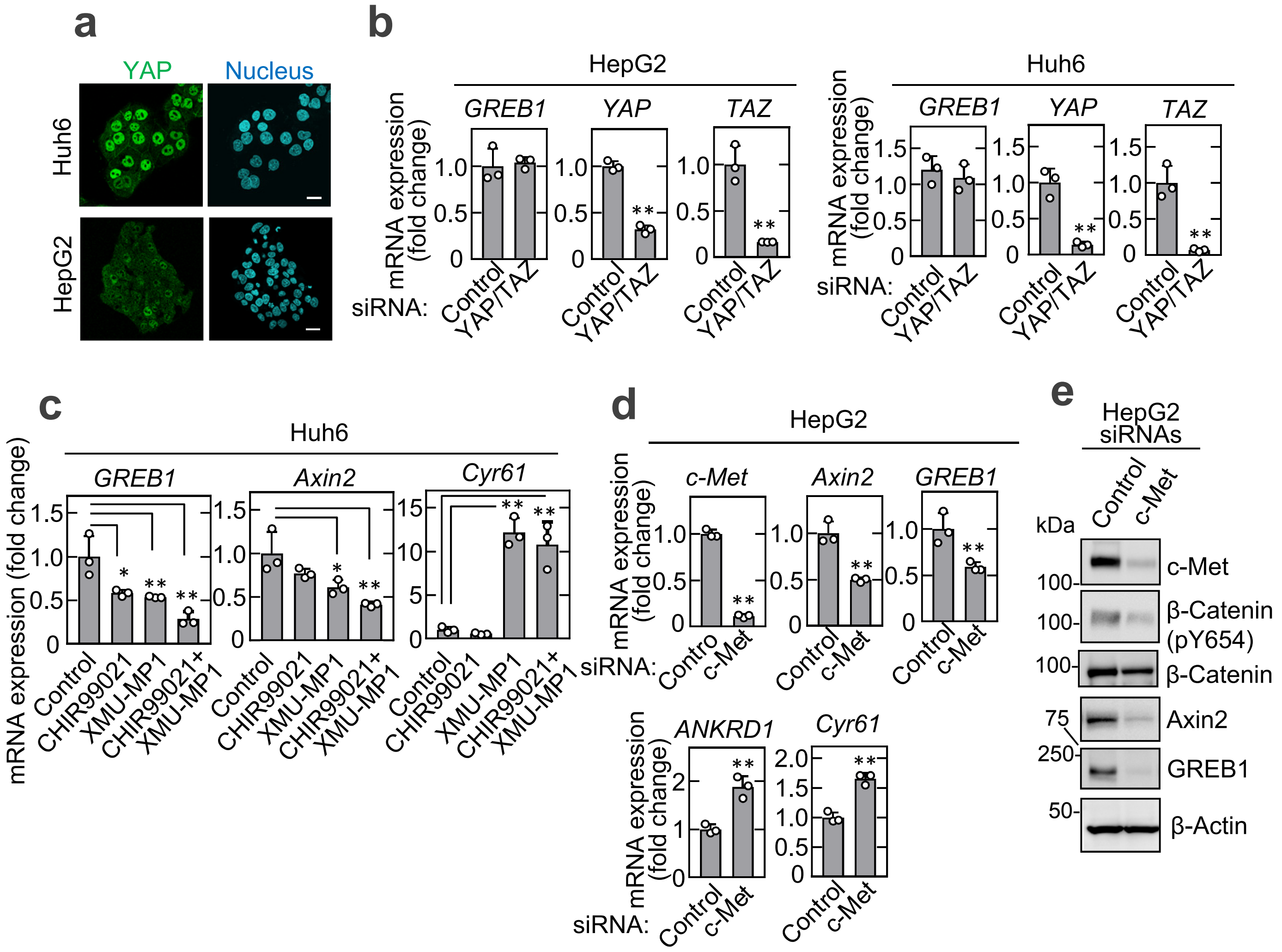

Supplementary Fig. 7| GREB1 expression is dependent on β-catenin and c-Met in HB cells.

a, Huh6 and HepG2 cells were stained with YAP antibody and Hoechst33342. **b**, HepG2 and Huh6 cells were transfected with control or *YAP/TAZ* siRNAs and real-time PCR analyses for the expression of the indicated mRNAs were performed. Results are expressed as fold changes compared with control cells and are shown as means ± SD. **c**, Huh6 cells were treated with CHIR99021 and/or XMU-MP1 for 24 hours and real-time PCR analyses for expression of the indicated mRNAs were performed. **d**, HepG2 cells were transfected with control or *c-Met* siRNA and real-time PCR analyses for expression of the indicated mRNAs were performed. **e**, HepG2 cells were transfected with control or *c-Met* siRNA and the lysates were probed with the indicated antibodies. ** $P<0.01$; * $P<0.05$, *t* test in **b** and **d**; ANOVA and post hoc test in **c**. Scale bar in **a**, 20 μm.

Supplementary Fig. 8

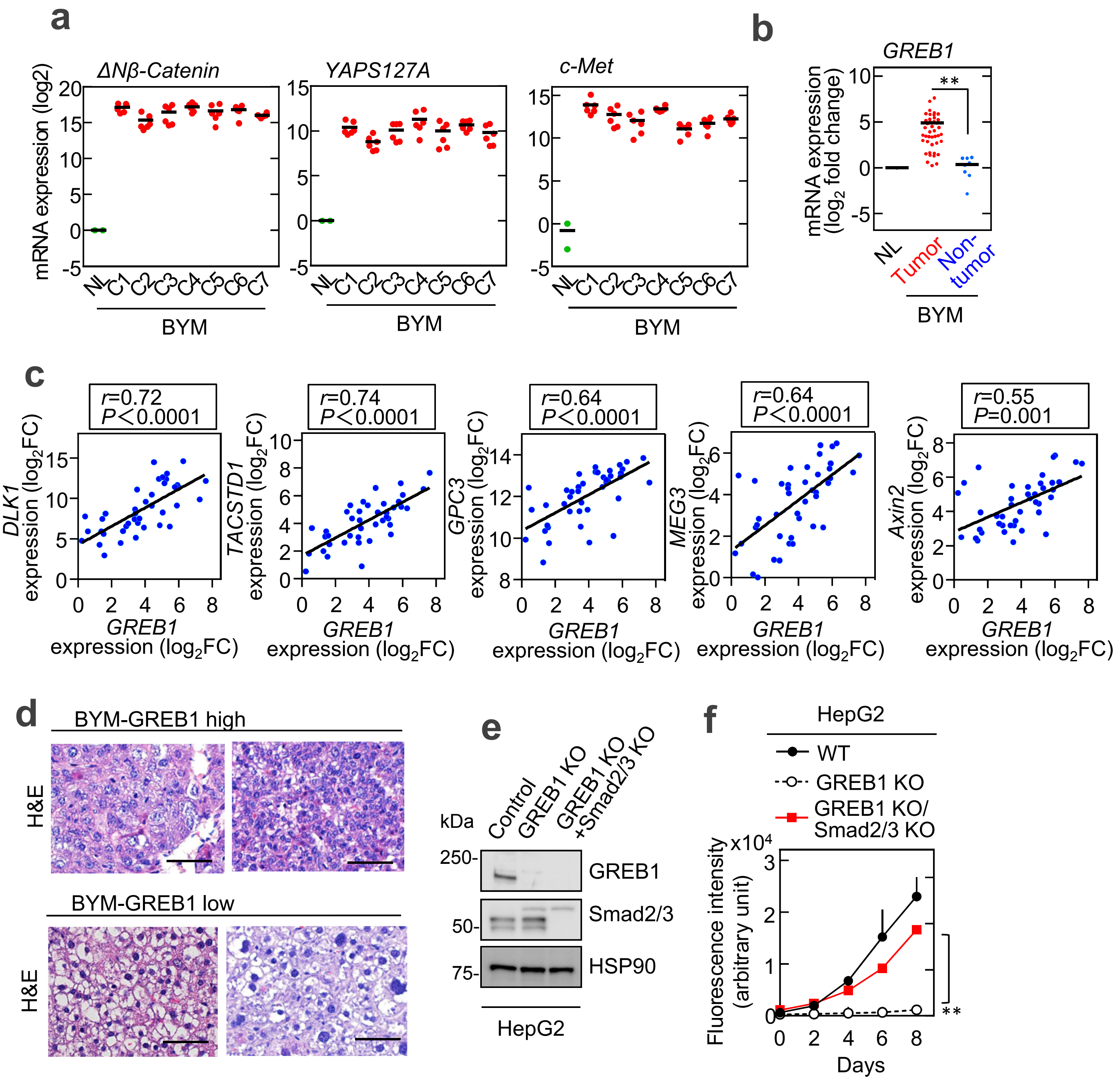

Supplementary Fig. 8| GREB1 is expressed in HB-like tumor induced by BYM.

a, Total RNAs were prepared from normal livers (NLs) (n=2) from WT mice and tumor nodules (n=42) from seven BYM mice. Real-time PCR analyses for expression of exogenous *ΔNβ-catenin*, *YAPS127A*, and *c-Met* mRNAs were performed. Results are expressed as $\log_2$ fold-changes compared with NLs and are shown as dots plot with median. **b**, Total RNAs were prepared from NL (n=8) from six WT mice and tumor nodules (n=42) and non-tumor tissues (n=8) from seven BYM mice. Real-time PCR experiments for *GREB1* mRNA expression are performed. Results are expressed as $\log_2$ fold-changes compared with NL and are shown as dots plot with median. **c**, Total RNA was prepared from six tumor nodules from seven BYM mice (n=42) and real-time PCR experiments for mRNA expression of the indicated genes were performed. Results expressed as $\log_2$ fold changes (FC) compared with NLs from untreated mice, are shown as scatter plots indicating correlation between GREB1 (X-axis) and HB-related genes (Y-axis). The solid line indicates linear fit; r indicates Pearson's correlation coefficient. *r* value and *P* value were calculated with GraphPad Prism 7. **d**, Tissue sections of the liver isolated from BYM mice with a high expression of GREB1 (C1 and C6) and with a low expression of GREB1 (C5) were stained with hematoxylin and eosin. **e**, Lysates of control, GREB1 knockout (KO), or GREB1 and Smad2/3 KO HepG2 cells were probed with anti-GREB1, anti-Smad2/3, and anti-HSP90 antibodies. **f**, Control, GREB1 knockout (KO), or GREB1 and Smad2/3 KO HepG2 cells were cultured on a 2D plastic dish for the indicated numbers of days. Relative number of cells were quantified using the Cyquant assay. ** $P<0.01$; * $P<0.05$, *t* test in **b**; ANOVA and post hoc test in **f**. Scale bars in **d**, 50 μm.

Supplementary Fig. 9

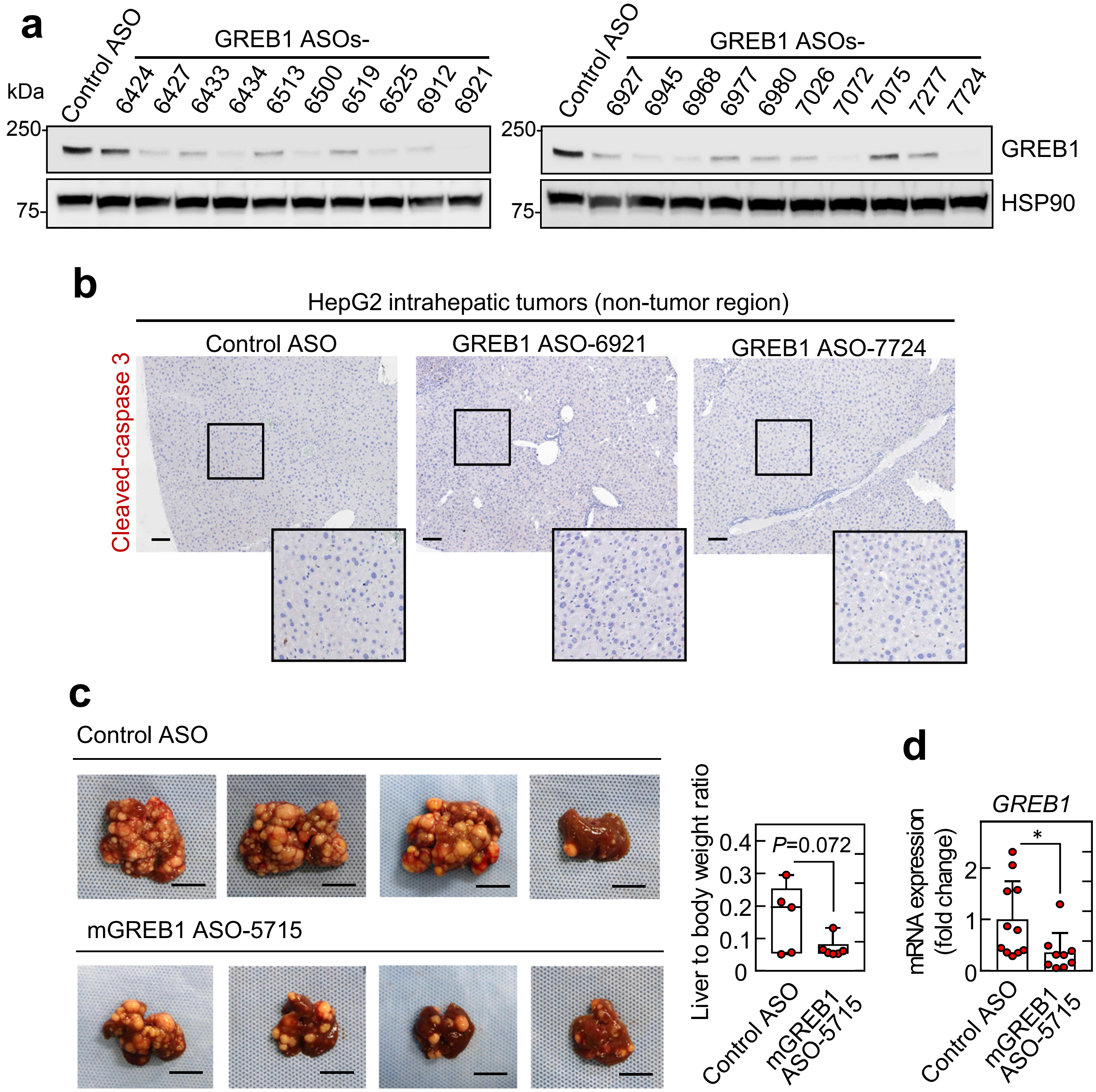

Supplementary Fig. 9| GREB1 ASOs inhibit GREB1 expression and liver tumor formation.

a, HepG2 cells transfected with control ASO or the indicated GREB1 ASOs and the lysates were probed with anti-GREB1 and anti-HSP90 antibodies. **b,** Sections from non-tumor regions in the liver with HepG2-induced tumors treated with control or GREB1 ASOs, were stained with anti-cleaved caspase 3 antibody and hematoxylin. Solid squares show enlarged images. **c,** ΔNβ-catenin, YAPS127A, and c-Met (BYM) plasmids were hydorodynamically injected into mice on day 0. Starting from day 3, control ASO (n = 5) or mouse GREB1 (mGREB1) ASO-5715 (n = 6) were administered subcutaneously twice a week. Representative liver images of the liver tumors at 6-7 weeks after hydrodynamic tail vein injection of plasmids are shown. Weights of the liver and whole body in BYM mice injected with ASOs were measured and the results are expressed as the ratio of liver weight to body weight. Results are shown as box plots. **d,** Real-time PCR analyses for *GREB1* mRNA expression in liver tumors were performed. Relative GREB1 mRNA levels were expressed as fold-changes compared with levels in control ASO-injected tumors. * $P<0.05$, *t* test in **d**. Scale bars in **b**, 100 μm; in **c**, 1 cm.

Supplementary Table 1

Supplementary Table 1| List of 11 genes which shows a significant decrease in β-catenin-depleted HepG2 cells.

Rank	Gene symbol	EntrezGene ID	Fold change (KD/control)	P-*value*
1	*NKD1*	85407	-12.814	.0000114
2	*LGR5*	8549	-9.022	.0000057
3	*TNFRSF19*	55504	-6.54	.00000068
4	*SP5*	389058	-5.755	.000165
5	*ZNRF3*	84133	-4.951	.000254
6	*RNF43*	54894	-4.191	.0000245
7	*AXIN2*	8313	-3.702	.0000362
8	*GREB1*	9687	-3.620	.000114
9	*CCND1*	595	-3.518	.0000273
10	*DKK1*	22943	-3.502	.000273
11	*LYZ*	4069	-3.037	.00000142

Supplementary Table 2

Supplementary Table 2| Characteristics of hepatoblastoma patients.

Patient ID	Age (year)	Sex	GREB1 expression	β-Catenin expression	YAP expression	Pathology	PRETEXT
#1	1	male	High	High	High	Combined fetal and embryonal	Ⅱ
#2	16	male	Low	High	High	Embryonal	Recurrence
#3	3	male	High	High	High	Combined fetal and embryonal	Ⅳ
#4	0	male	Negative	Negative	Negative	Fetal	Ⅲ
#5	8	female	Low	High	High	Combined fetal and embryonal	Ⅳ
#6	9	female	Low	High	High	Embryonal	Ⅳ
#7	0	male	Low	High	High	Fetal	Ⅲ
#8	2	male	High	High	High	Embryonal	Ⅳ
#9	2	female	High	High	High	Fetal	Ⅲ
#10	4	male	High	Negative	Negative	Combined fetal and embryonal	Ⅲ
#11	2	female	Low	High	High	Fetal	Ⅲ

Supplementary Table 3

Supplementary Table 3| Candidates of GREB1 binding proteins in BioGRID database.

	Interactor (Bait)	Experiment	Dataset (PMID)
1	*BCAR3*	*Two-hybrid*	25640309
2	*CASP8*	*Two-hybrid*	25640309
3	*CDKN2C*	*Two-hybrid*	25640309
4	*DNAJC7*	*Affinity capture-MS*	28514442
5	*DPPA3*	*Affinity capture-MS*	Pre-publication
6	*HMMR*	*Two-hybrid*	25640309
7	*HRAS*	*Two-hybrid*	25640309
8	*LSP1*	*Two-hybrid*	25640309
9	*PALB2*	*Two-hybrid*	25640309
10	*RBCC1*	*Two-hybrid*	25640309
11	*SMAD4*	*Two-hybrid*	25640309
12	*TGFβ1*	*Two-hybrid*	25640309
13	*TKT*	*Affinity capture-MS*	28514442
14	*TTC9C*	*Affinity capture-MS*	Pre-publication
15	*XRCC3*	*Two-hybrid*	25640309

Supplementary Table 4

Supplementary Table 4| The correlation analyses among HB markers in the tumors of BMY mice.

	TACSTD1	*GPC*	*AFP*	*MEG3*	*BEX1*	*PEG3*	*Axin2*	*DLK1*
GREB1	0.75	0.65	0.70	0.55	0.68	0.40	0.55	0.72
TACSTD1		0.60	0.47	0.34	0.58	0.31	0.48	0.43
GPC			0.81	0.60	0.76	0.39	0.62	0.62
AFP				0.79	0.83	0.62	0.44	0.80
MEG3					0.65	0.73	0.39	0.78
BEX1						0.71	0.33	0.69
PEG3							0.13	0.55
Axin2								0.50

The values indicate a correlation coefficient.

Supplementary Table 5| Sequences of GREB1 ASOs used in this study.

No.	GREB1 ASOs	Sequences (5'→3')
1	hGREB1 ASO-6424	G(Y)^A(Y)^5(Y)^a^g^g^a^a^g^t^a^a^A(Y)^T(Y)^c
2	hGREB1 ASO-6427	5(Y)^A(Y)^G(Y)^g^a^c^a^g^g^a^a^g^T(Y)^A(Y)^a
3	hGREB1 ASO-6433	G(Y)^A(Y)^A(Y)^t^g^g^c^a^g^g^a^c^A(Y)^G(Y)^g
4	hGREB1 ASO-6434	5(Y)^G(Y)^A(Y)^a^t^g^g^c^a^g^g^a^5(Y)^A(Y)^g
5	hGREB1 ASO-6500	G(Y)^5(Y)^T(Y)^a^c^a^a^a^a^t^a^a^5(Y)^5(Y)^c
6	hGREB1 ASO-6513	A(Y)^A(Y)^A(Y)^t^a^c^t^g^g^c^a^c^5(Y)^G(Y)^c
7	hGREB1 ASO-6519	5(Y)^T(Y)^A(Y)^c^t^g^a^a^a^t^a^c^T(Y)^G(Y)^g
8	hGREB1 ASO-6525	T(Y)^5(Y)^5(Y)^c^a^t^c^t^a^c^t^g^A(Y)^A(Y)^a
9	hGREB1 ASO-6912	A(Y)^A(Y)^G(Y)^t^a^a^g^c^a^a^t^g^T(Y)^G(Y)^g
10	hGREB1 ASO-6921	G(Y)^T(Y)^5(Y)^t^g^t^t^t^c^a^a^g^T(Y)^A(Y)^a
11	hGREB1 ASO-6927	T(Y)^T(Y)^5(Y)^a^t^t^g^t^c^t^g^t^T(Y)^T(Y)^c
12	hGREB1 ASO-6945	T(Y)^A(Y)^T(Y)^a^t^c^a^c^t^t^t^g^G(Y)^T(Y)^t
13	hGREB1 ASO-6968	T(Y)^5(Y)^T(Y)^a^g^t^t^c^t^c^a^t^5(Y)^A(Y)^a
14	hGREB1 ASO-6977	A(Y)^G(Y)^T(Y)^c^a^t^a^a^g^t^c^t^A(Y)^G(Y)^t
15	hGREB1 ASO-6980	T(Y)^A(Y)^5(Y)^a^g^t^c^a^t^a^a^g^T(Y)^5(Y)^t
16	hGREB1 ASO-7026	G(Y)^A(Y)^G(Y)^a^a^a^a^t^g^a^g^c^T(Y)^A(Y)^c
17	hGREB1 ASO-7072	T(Y)^5(Y)^T(Y)^c^t^a^g^t^c^a^a^g^T(Y)^G(Y)^a
18	hGREB1 ASO-7075	T(Y)^5(Y)^5(Y)^t^c^t^c^t^a^g^t^c^A(Y)^A(Y)^g
19	hGREB1 ASO-7277	G(Y)^A(Y)^G(Y)^a^a^t^g^g^t^g^a^g^A(Y)^A(Y)^c
20	hGREB1 ASO-7724	A(Y)^T(Y)^T(Y)^g^a^g^g^g^t^a^g^g^5(Y)^A(Y)^a
21	mGREB1 ASO-5715	5(Y)^5(Y)^G(Y)^a^g^c^a^g^g^g^c^a^T(Y)^A(Y)^g
22	Control ASO	T(Y)^a^g^A(Y)^g^a^G(Y)^t^a^5(Y)^c^c^A(Y)^t^c

Lower Case=DNA / N(Y)=AmNA / 5(Y)=AmNA_mC / ^=Phosphorothioated
