# Supplementary material for: GREB1 induced by Wnt signaling promotes development of hepatoblastoma by suppressing TGFβ signaling
Source: Nat Commun. 2019 Aug 28;10:3882. doi: 10.1038/s41467-019-11533-x (PMC6713762; doi:10.1038/s41467-019-11533-x)
Supplement: Supplementary file 3 — Reporting Summary [file 41467_2019_11533_MOESM3_ESM.pdf]

nature research

Corresponding author(s): Akira Kikuchi (NCOMMS-18-31601)

Last updated by author(s): May 29, 2019

Reporting Summary

Nature Research wishes to improve the reproducibility of the work that we publish. This form provides structure for consistency and transparency in reporting. For further information on Nature Research policies, see Authors & Referees and the Editorial Policy Checklist.

Statistics

For all statistical analyses, confirm that the following items are present in the figure legend, table legend, main text, or Methods section.

n/a	Confirmed	
☐	☒	The exact sample size (*n*) for each experimental group/condition, given as a discrete number and unit of measurement
☐	☒	A statement on whether measurements were taken from distinct samples or whether the same sample was measured repeatedly
☐	☒	The statistical test(s) used AND whether they are one- or two-sided<br>*Only common tests should be described solely by name; describe more complex techniques in the Methods section.*
☒	☐	A description of all covariates tested
☐	☒	A description of any assumptions or corrections, such as tests of normality and adjustment for multiple comparisons
☐	☒	A full description of the statistical parameters including central tendency (e.g. means) or other basic estimates (e.g. regression coefficient) AND variation (e.g. standard deviation) or associated estimates of uncertainty (e.g. confidence intervals)
☐	☒	For null hypothesis testing, the test statistic (e.g. *F*, *t*, *r*) with confidence intervals, effect sizes, degrees of freedom and *P* value noted<br>*Give P values as exact values whenever suitable.*
☒	☐	For Bayesian analysis, information on the choice of priors and Markov chain Monte Carlo settings
☒	☐	For hierarchical and complex designs, identification of the appropriate level for tests and full reporting of outcomes
☐	☒	Estimates of effect sizes (e.g. Cohen's *d*, Pearson's *r*), indicating how they were calculated

Our web collection on statistics for biologists contains articles on many of the points above.

Software and code

Policy information about availability of computer code

Data collection	Image Studio Software (v2.1.10, LI-COR Bioscience, Bad Homburg, Germany), StepOne Software Real-Time PCR System (ThermoFisher Scientific, Offenbach, Germany), Image- Quant LAS 4000 detection system (GE Healthcare, Munich, Germany), LSM Image Browser (Carl Zeiss, Jena, Germany), Zen software (Carl Zeiss), cellSens software (Olympus, Tokyo, Japan).
Data analysis	Excel 2010 (Microsoft, Redmond, WA, USA), GraphPad Prism 7 (GraphPad Software, Inc, La Jolla, CA, USA),

For manuscripts utilizing custom algorithms or software that are central to the research but not yet described in published literature, software must be made available to editors/reviewers. We strongly encourage code deposition in a community repository (e.g. GitHub). See the Nature Research guidelines for submitting code & software for further information.

Data

Policy information about availability of data

All manuscripts must include a data availability statement. This statement should provide the following information, where applicable:

- Accession codes, unique identifiers, or web links for publicly available datasets
- A list of figures that have associated raw data
- A description of any restrictions on data availability

The data that support the findings of this study are available from the corresponding author upon reasonable request.

Field-specific reporting

Please select the one below that is the best fit for your research. If you are not sure, read the appropriate sections before making your selection.

☒ Life sciences ☐ Behavioural & social sciences ☐ Ecological, evolutionary & environmental sciences

For a reference copy of the document with all sections, see nature.com/documents/nr-reporting-summary-flat.pdf

Life sciences study design

All studies must disclose on these points even when the disclosure is negative.

Sample size	Sample sizes were determined depending on the experimental settings and availability of the biospecimen. For in vivo experiments, we were limited to certain sample sizes due to the animal proposal.
Data exclusions	Data were excluded where the investigator noted an technical error that may have affected results.
Replication	Attempts to replicate the experiments were successful.
Randomization	No randomization was conducted for the in vitro samples or animal samples used in our study.
Blinding	In vitro and in vivo studies were not performed in a blinded fashion due to the high risk of confusion in handling of the samples by different experimenters.

Reporting for specific materials, systems and methods

We require information from authors about some types of materials, experimental systems and methods used in many studies. Here, indicate whether each material, system or method listed is relevant to your study. If you are not sure if a list item applies to your research, read the appropriate section before selecting a response.

Materials & experimental systems

n/a	Involved in the study	
☐	☒	Antibodies
☐	☒	Eukaryotic cell lines
☒	☐	Palaeontology
☐	☒	Animals and other organisms
☐	☒	Human research participants
☒	☐	Clinical data

Methods

n/a	Involved in the study	
☒	☐	ChIP-seq
☒	☐	Flow cytometry
☒	☐	MRI-based neuroimaging

Antibodies

Antibodies used

All antibodies were purchased as follows:
Anti-GREB1 (#MABS62) , anti-phospho-HistoneH3 (ser10) (#06-570), and anti-acetyl-Histone H4 (#06-866) antibodies were from Merck Millipore (Billerica, MA, USA).
Anti-HSP90 (#610419), anti-Smad2/3 (#610842), anti-β-catenin (#610154), and anti-N-cadherin (#610920) antibodies were from BD Biosciences (San Jose, CA, USA).
Anti-GREB1 (#sc-138794), anti-Smad4 (#sc-7966), anti-p300 (#sc-585), and anti-GFP (#sc-9996) antibodies were purchased from Santa Cruz Santa Cruz Biotechnology, (Dallas, TX, USA).
Anti-Smad2/3 (#8685) , anti-phospho-Smad2 (Ser465/467)/Smad3 (Ser423/425) (#8828), anti-cleaved caspase3 (#9661), anti-PARP (#9532), anti-HistoneH3 (#9715), Ki-67 (#9027), anti-YAP1 (#14074), anti-c-Met (#8198), anti-c-Myc (#5605), anti-TGFβ (#3711), and anti-Axin2 (#2151) antibodies were from Cell Signaling Technology (Beverly, MA, USA).
Anti-β-tubulin (#T8328), anti-β-actin (#A5316), anti-phospho-Catenin-β (pTyr654) (#SAB4504128) antibodies were from Sigma-Aldrich (Steinheim, Germany).
Anti-GFP (#A6455) antibody was from Life Technologies/Thermo Fisher Scientific (Carlsbad, CA, USA).
Anti-Smad2/3 (#ab207447) antibody was from Abcam (Cambridge, UK).
and anti-FLAG (#014-22383) antibody was from WAKO (Tokyo, Japan).
Anti-DLK1 (#10636-1-AP) antibody was from Proteintech Group, Inc (Chicago, IL, USA).
Anti-DLK1 (#MAB8634) antibody was from R&D Systems, (Minneapolis, MN, USA).

Validation

Antibodies are commercially available and validated in the literature as cited on the manufacturers websites, as well as by the manufacturers data sheets .

Eukaryotic cell lines

Policy information about cell lines

Cell line source(s)	HepG2 cells were purchased from American Type Culture Collection (ATCC, Manassas, VA, USA). MCF7, HLE, and Huh7 cells were from Japanese Collection of Research Bioresources (JCRB, Osaka, Japan). Lenti-XTM293T (X293T) cells were from Takara Bio Inc. (Shiga, Japan). Huh6 hepatoblastoma cells were kindly provided by Dr. H. Okuyama (Osaka University, Suita, Japan) in August 2015. SNU387, SNU449, and BMEL cells were kindly provided by Dr. T. Kodama (Osaka University, Suita, Japan) in January 2017.
Authentication	Authentication was provided by ATCC or JCRB. Other cell lines were not authenticated.
Mycoplasma contamination	The cell lines were not tested for mycoplasma contamination.
Commonly misidentified lines (See ICLAC register)	There are no misidentified cell lines in this study.

Animals and other organisms

Policy information about studies involving animals; ARRIVE guidelines recommended for reporting animal research

Laboratory animals	Mice used in the experiments were 6-8 weeks old male mice.<br>C57BL6/N mice (Japan SLC Inc., Hamamatsu, Japan),<br>BALB/cAJcl-nu/nu mice of 5-weeks old (nude mice; CLEA Japan, Tokyo, Japan)
Wild animals	The study did not involve wild animals.
Field-collected samples	The study did not involve samples collected from field.
Ethics oversight	The Animal Research Committee of Osaka University, Japan (No.26-032-048).

Note that full information on the approval of the study protocol must also be provided in the manuscript.

Human research participants

Policy information about studies involving human research participants

Population characteristics	The total number of HB patients is 11. The ages of the patients ranged from 0 to 16 years (median, 3 years). The detailed patient clinical information can be found in the Supplementary Table 2.
Recruitment	Participants were patients who had undergone surgery at Osaka University Hospital from January 2008 to March 2015. All samples were obtained under protocols approved by the ethical review board of the Graduate School of Medicine, Osaka University, Japan (No. 13455).
Ethics oversight	The ethical review board of the Graduate School of Medicine, Osaka University, Japan (No. 13552).

Note that full information on the approval of the study protocol must also be provided in the manuscript.
