# Supplementary material for: GREB1 induced by Wnt signaling promotes development of hepatoblastoma by suppressing TGFβ signaling
Source: Nat Commun. 2019 Aug 28;10:3882. doi: 10.1038/s41467-019-11533-x (PMC6713762; doi:10.1038/s41467-019-11533-x)
Supplement: Supplementary file 4 — Source Data [file 41467_2019_11533_MOESM4_ESM.pdf]

Fig 1c

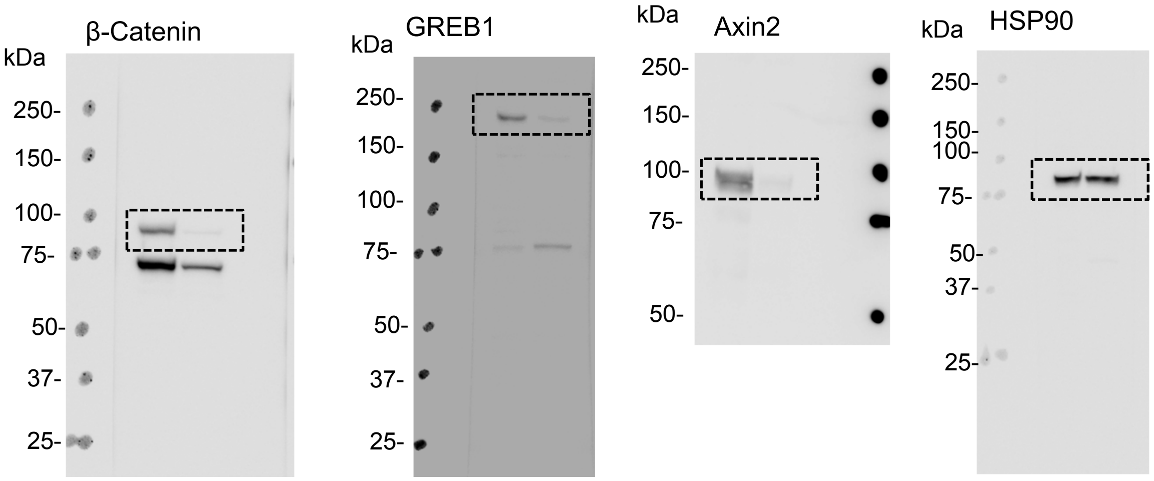

Source data 1. Uncropped western blots for Fig 1.
Dotted lines indicate cropped area.

Fig 2d

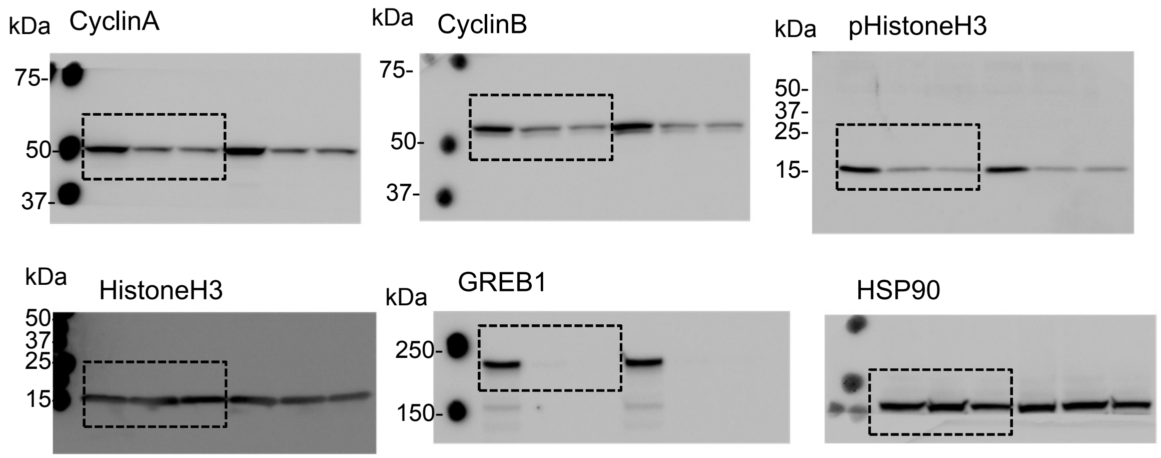

Fig 2g

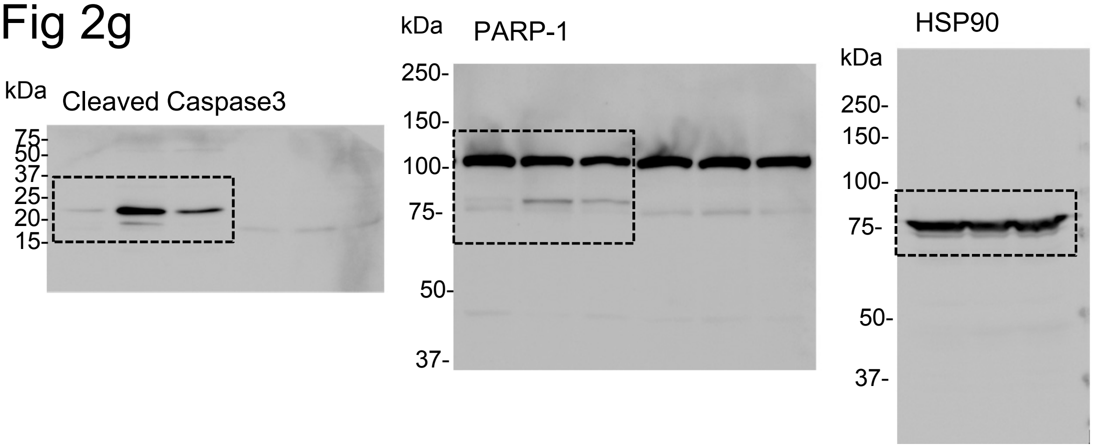

Source data 2. Uncropped western blots for Fig 2.
Dotted lines indicate cropped area.

Fig 3b

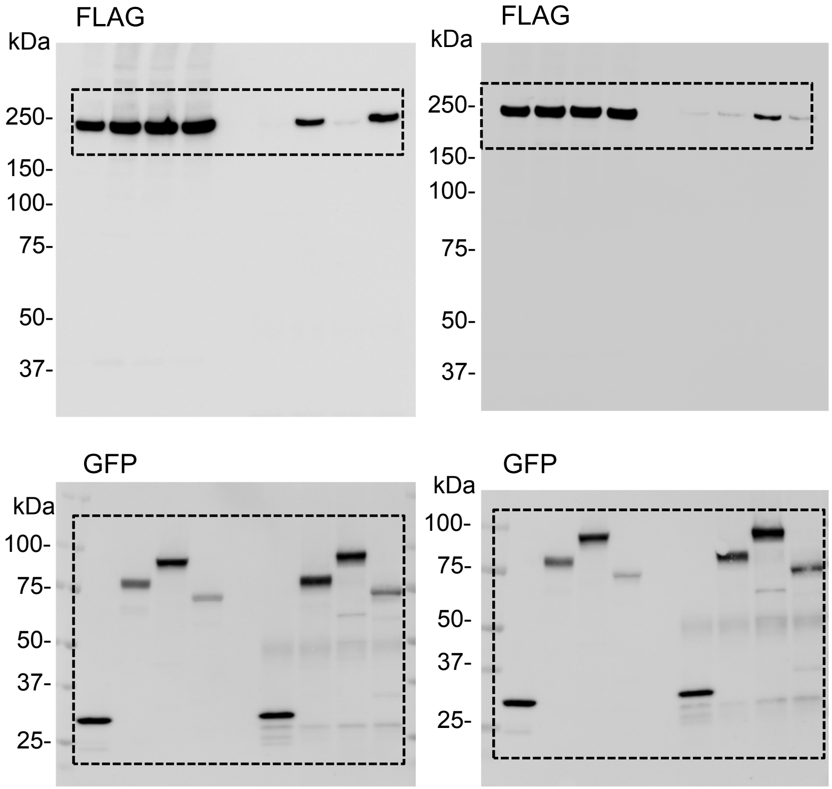

Fig 3c

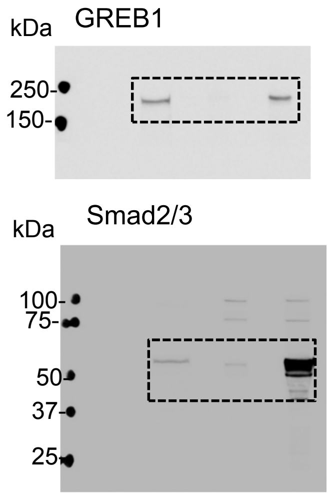

Fig 3d

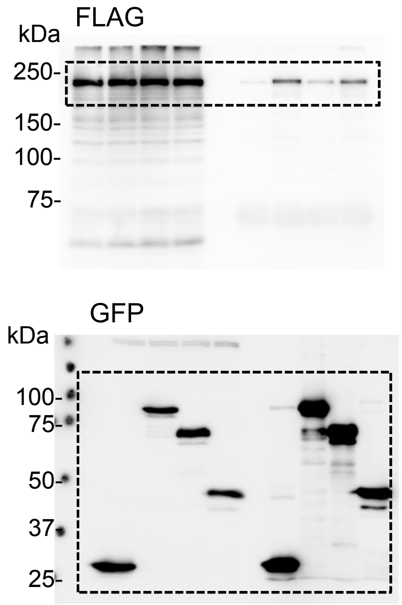

Fig 3e

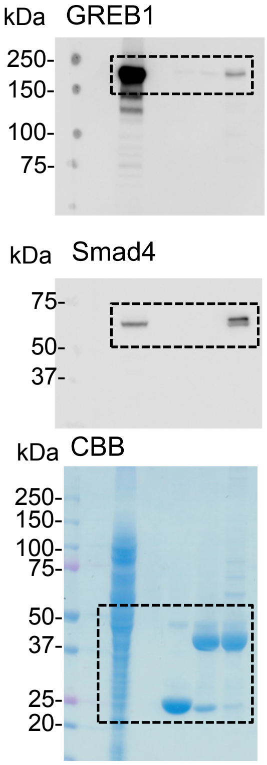

Fig 3g

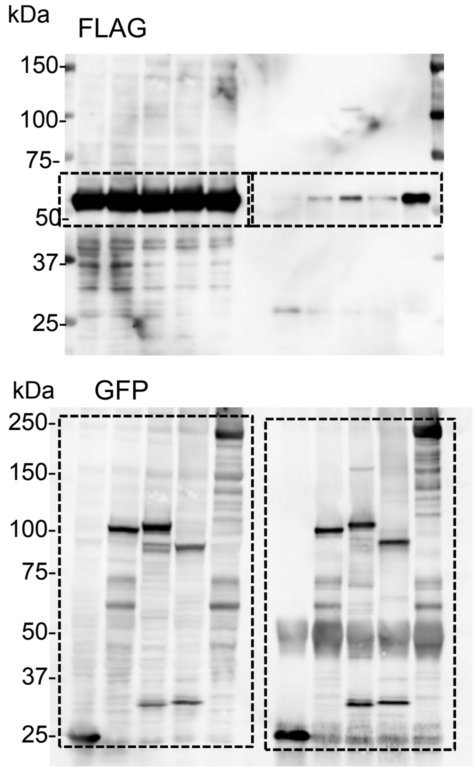

Source data 3. Uncropped western blots for Fig 3. Dotted lines indicate cropped area.

Fig 4e

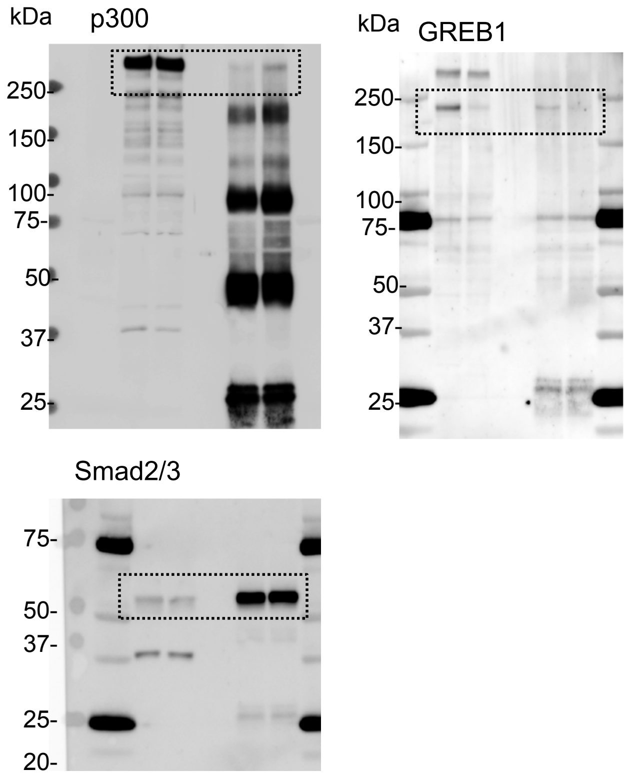

Fig 4f

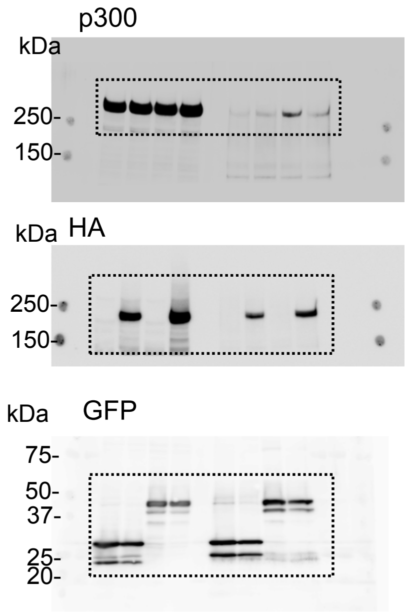

Source data 4. Uncropped western blots for Fig 4.
Dotted lines indicate cropped area.

SFig 2b

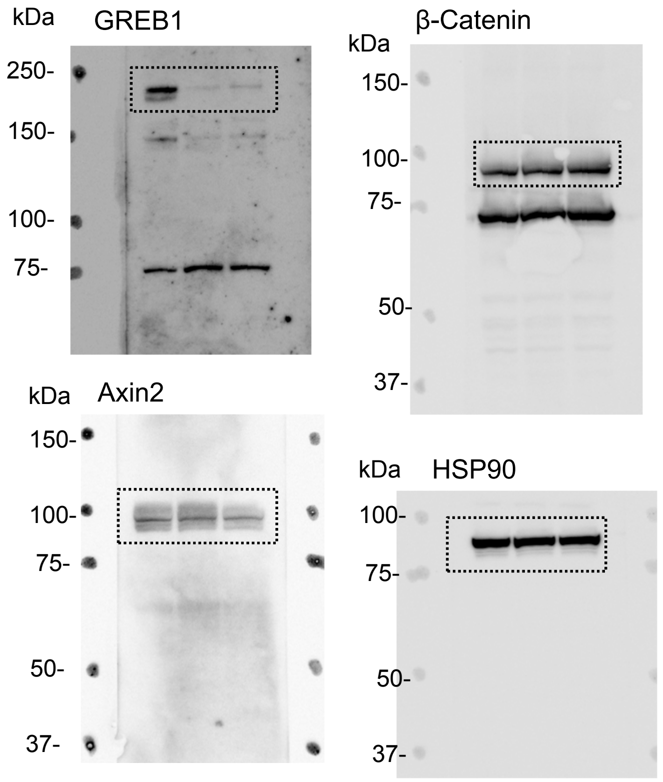

SFig 2g

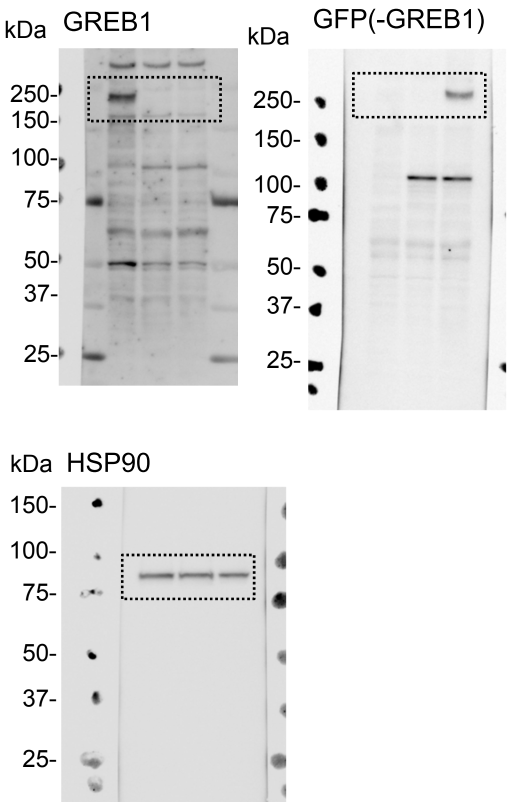

SFig 2j

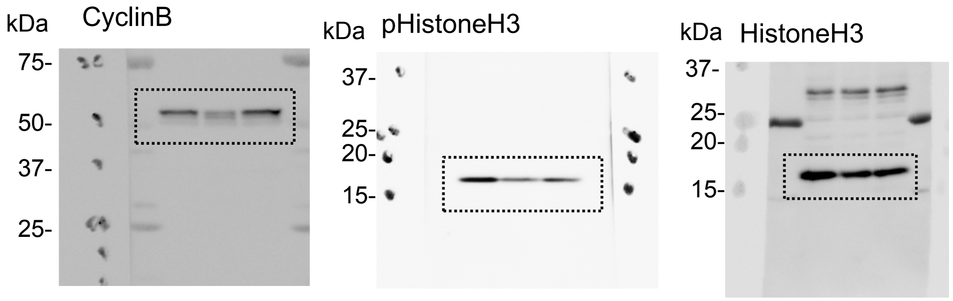

Source data 5. Uncropped western blots for Supplementary Fig 2. Dotted lines indicate cropped area.

SFig 3b

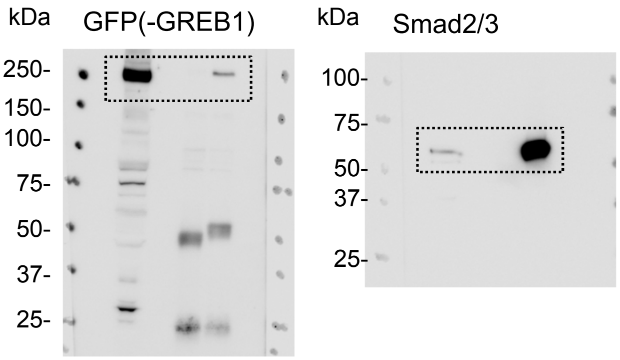

SFig 3c

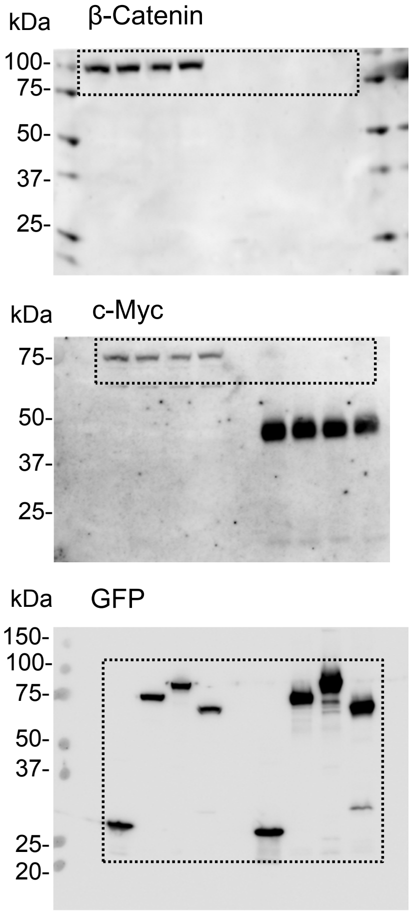

SFig 3d

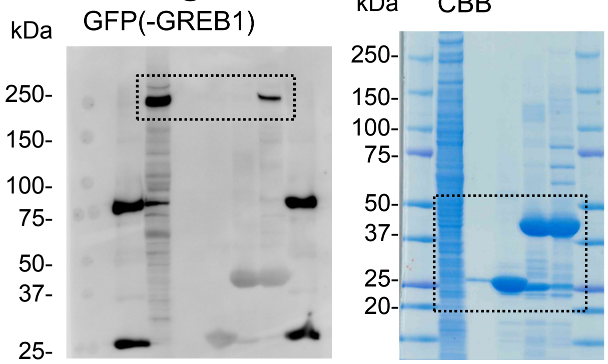

SFig 3e

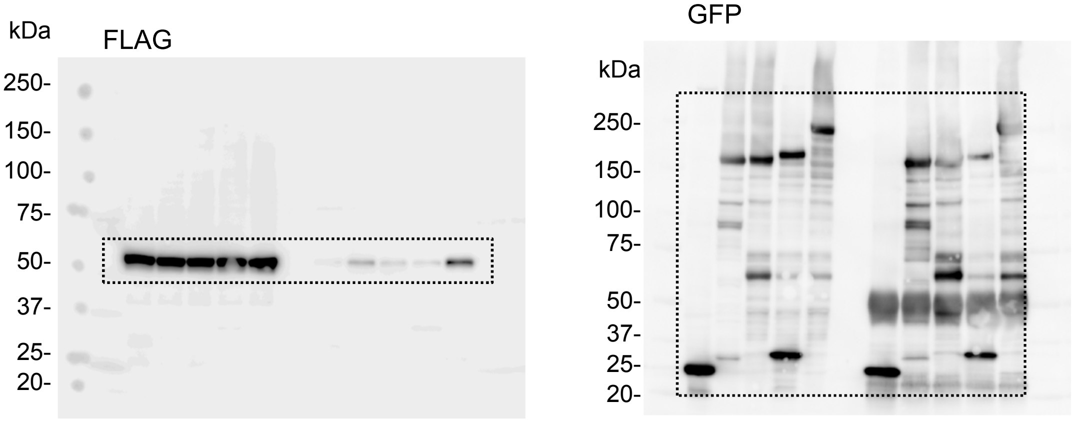

Source data 6. Uncropped western blots for Supplementary Fig 3. Dotted lines indicate cropped area.

SFig 4d

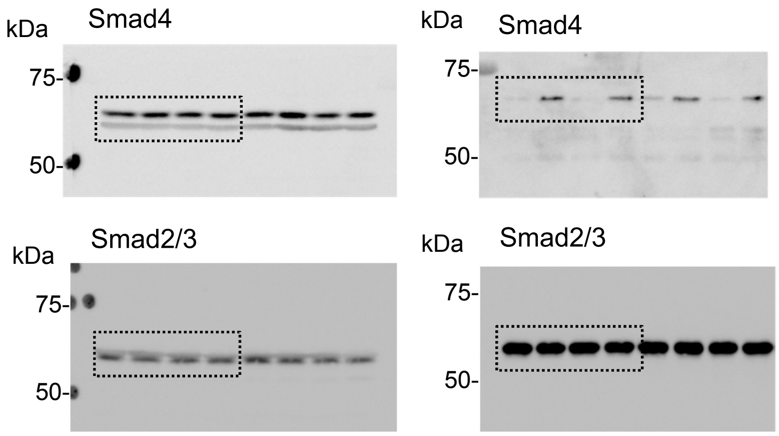

SFig 4e

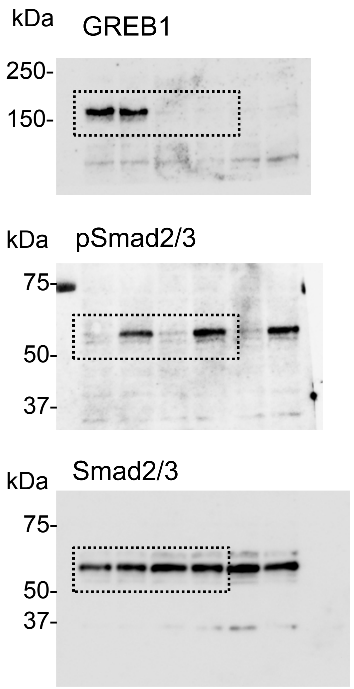

SFig 4g

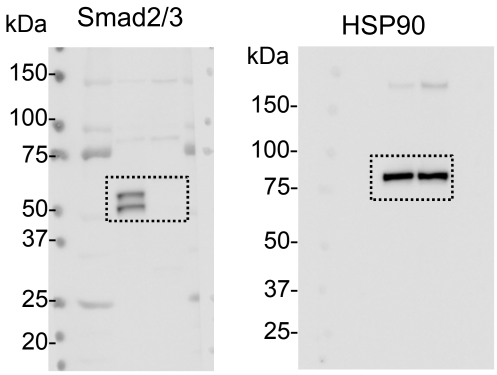

Source data 7. Uncropped western blots for Supplementary Fig 4. Dotted lines indicate cropped area.

SFig 5c

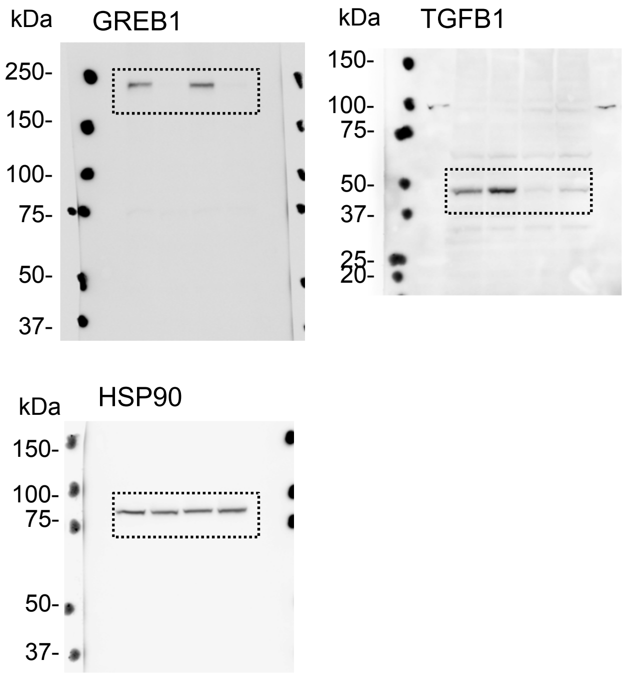

SFig 5k

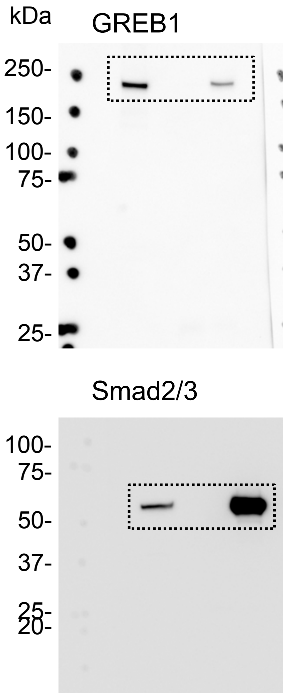

Source data 8. Uncropped western blots for Supplementary Fig 5. Dotted lines indicate cropped area.

SFig 7e

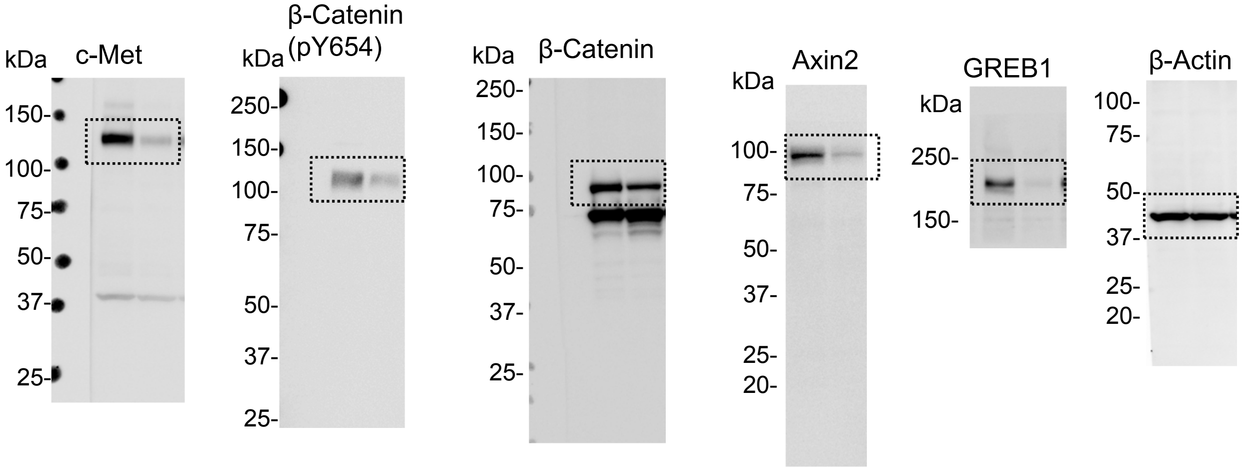

Source data 9. Uncropped western blots for Supplementary Fig 7. Dotted lines indicate cropped area.

SFig 8e

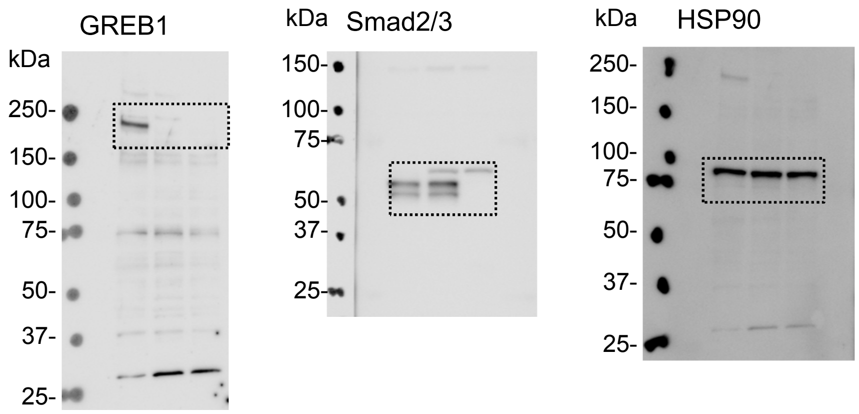

Source data 10. Uncropped western blots for Supplementary Fig 8. Dotted lines indicate cropped area.

SFig 9a

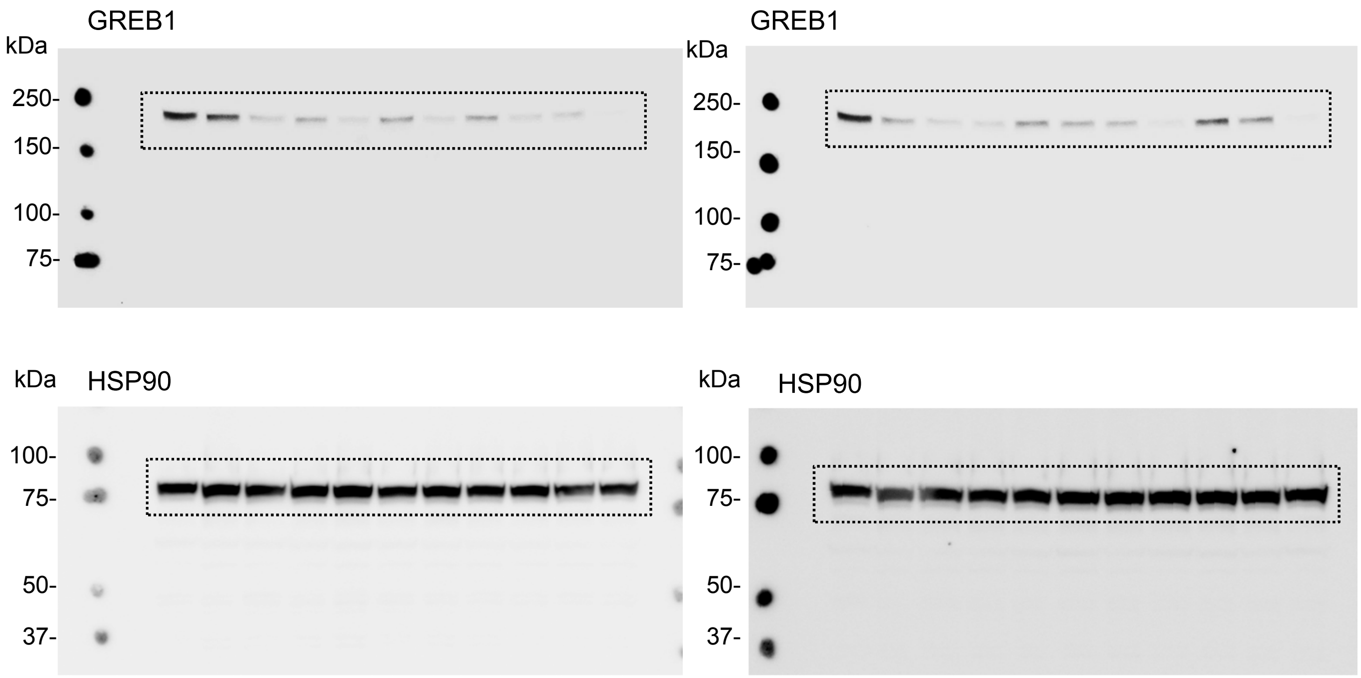

Source data 11. Uncropped western blots for Supplementary Fig 9. Dotted lines indicate cropped area.
